# A direct detection of *Escherichia coli *genomic DNA using gold nanoprobes

**DOI:** 10.1186/1477-3155-10-8

**Published:** 2012-02-06

**Authors:** BakthavathsalamPadmavathypadmavathy.s.b@gmail.com, RajendranVinoth Kumark.r.vinoth85@gmail.com, Baquir MohammedJaffar Alijaffarali.bm@gmail.com

**Affiliations:** 1 AU-KBC Research Centre, M.I.T.Campus of Anna University, Tamil Nadu, India; 2 Centre for Green Energy Technology, Pondicherry University, Puducherry, India

**Keywords:** Gold nanoparticles, DNA detection, colorimetric detection, clinical diagnosis, *Escherichia coli*

## Abstract

**Background:**

In situation like diagnosis of clinical and forensic samples there exists a need for highly sensitive, rapid and specific DNA detection methods. Though conventional DNA amplification using PCR can provide fast results, it is not widely practised in diagnostic laboratories partially because it requires skilled personnel and expensive equipment. To overcome these limitations nanoparticles have been explored as signalling probes for ultrasensitive DNA detection that can be used in field applications. Among the nanomaterials, gold nanoparticles (AuNPs) have been extensively used mainly because of its optical property and ability to get functionalized with a variety of biomolecules.

**Results:**

We report a protocol for the use of gold nanoparticles functionalized with single stranded oligonucleotide (AuNP- oligo probe) as visual detection probes for rapid and specific detection of *Escherichia coli*. The AuNP- oligo probe on hybridization with target DNA containing complementary sequences remains red whereas test samples without complementary DNA sequences to the probe turns purple due to acid induced aggregation of AuNP- oligo probes. The color change of the solution is observed visually by naked eye demonstrating direct and rapid detection of the pathogenic *Escherichia coli *from its genomic DNA without the need for PCR amplification. The limit of detection was ~54 ng for unamplified genomic DNA. The method requires less than 30 minutes to complete after genomic DNA extraction. However, by using unamplified enzymatic digested genomic DNA, the detection limit of 11.4 ng was attained. Results of UV-Vis spectroscopic measurement and AFM imaging further support the hypothesis of aggregation based visual discrimination. To elucidate its utility in medical diagnostic, the assay was validated on clinical strains of pathogenic *Escherichia coli *obtained from local hospitals and spiked urine samples. It was found to be 100% sensitive and proves to be highly specific without any cross reaction with non-*Escherichia coli *strains.

**Conclusion:**

This work gives entry into a new class of DNA/gold nanoparticles hybrid materials which might have optical property that can be controlled for application in diagnostics. We note that it should be possible to extend this strategy easily for developing new types of DNA biosensor for point of care detection. The salient feature of this approach includes low-cost, robust reagents and simple colorimetric detection of pathogen.

## Background

The development of highly sensitive and selective DNA detection methods is extremely important in clinical diagnosis, forensic investigations and gene therapy because the DNA is usually present at very low concentrations [1-3]. Although conventional DNA amplification using PCR can provide fast results, it is not widely used in the diagnostic laboratories of developing countries partially because it requires considerable skill and expensive equipment. To overcome these problems nanoparticles have been explored as signalling probes for ultrasensitive DNA detection that can be used in field applications. Among the nanomaterials, gold nanoparticles (AuNPs) have been extensively used for biomolecule detection by many research groups mainly because of its optical property and ability to functionalize with a variety of biomolecules. The AuNPs have been integrated in research and routine diagnostic applications and have been shown to have the great potential [4,5]. The colloidal AuNPs are used in the development of several biodetection schemes [6-10]. Although protein-coated gold colloids have been used extensively in lateral flow immunoassay based analytical techniques their application towards DNA detection has been introduced by Mirkin and co-workers [11]. The functionalization of colloidal AuNPs with alkyl thiol modified DNA is the most common ones. Such approach was used for preparing stable oligonucleotide conjugates with Au [12], Au-coated Ag [13] and ZnS-coated CdSe nanoparticles [14]. Considerable work was carried out that explore the basic properties of gold nanoparticles [15-17]. DNA-functionalized AuNPs are used in a variety of molecular diagnostic applications such as high sensitivity DNA detection in homogenous solution [18] and microarray- based DNA detection [19], lab-on-chip based substrates [20-22], atomic force microscopy based detection [23], detection of polymerase chain reaction (PCR) amplicons [24,25]. The optical property of colloidal AuNPs forms the basis of its application in rapid and specific hybridization based DNA detections. This property has already been applied for detection of eukaryotic gene expression [26], *Mycobacterium tuberculosis *detection [27,28] and for RNA quantification to detect chronic myeloid leukaemia [29]. Using a parallel approach we extend the application of AuNPs conjugated oligonucleotide probes (AuNP- oligo probes) as a tool for rapid detection of bacterial pathogen from clinical isolates. In this context, we demonstrated the utility of the assay on diarrhogenic and uropathogenic *Escherichia coli *strains obtained from patients of local hospitals.

**Figure 1:**
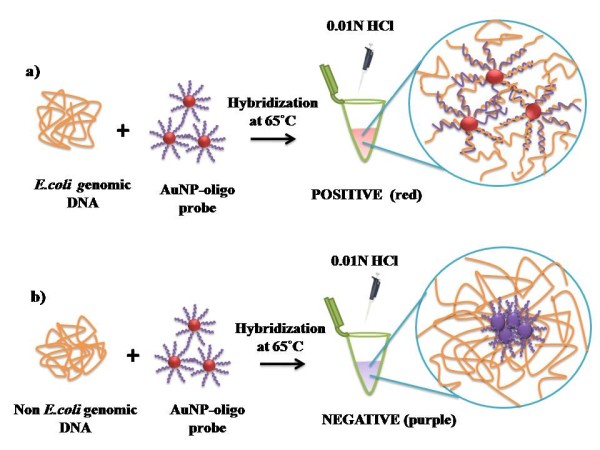
**Schematic representation of working of AuNP- oligo probe assay**. a) In the presence of *Escherichia coli *genomic DNA, AuNP- oligo probes are stabilized and prevented from aggregation upon acid addition. Red color of the solution indicates presence of target DNA and hence POSITIVE. b) In the presence of non *Escherichia coli *genomic DNA, AuNP- oligo probes loses its stability and tend to aggregate. Purple color of the solution indicates absence of target DNA and hence NEGATIVE.

Diagnosis of *Escherichia coli *infections requires bacterial culture that needs one to two days of incubation, and subsequent confirmatory testing. Rapid detection methods like enzyme immunoassay require a high population of the target pathogen [30,31]. In contrast, molecular diagnostic assays like PCR based approaches require fewer amounts of sample but dedicated equipments and trained technical personnel. Hence there exists always a need for rapid clinical diagnosis of pathogens at low cost. Our approach to using hybridization of oligonucleotides for the controlled assembly of gold nanoparticles under acidic environment for detection of *Escherichia coli *is outlined in Figure 1. In brief, the hybridization of the AuNP- oligo probe with the complementary *Escherichia coli *genomic DNA remains red. However the solution turns purple without complementary DNA. The method relies on a visual comparison of solutions for color change before and after acid induced aggregation of AuNP- oligo probe. Notably, the concomitant color change of the solution can be observed by naked eye. The assay was further evaluated by UV-Vis spectroscopic characterization, which demonstrates aggregation induced red-shift in AuNP- oligo probes. Additionally dispersion and aggregation pattern of AuNP- oligo probes respective to the hybridization was monitored at nanoscale using AFM. The application of the developed assay to medical diagnostics was shown a) clinical isolates and b) urine-spiked clinical samples. This study demonstrates the application of AuNP- oligo probes for visual detection of pathogen at genomic DNA level which can be adapted as a routine screening tool in clinical laboratories.

## Methods

### Bacterial strains and reagents

The uropathogenic *Escherichia coli *(UPEC) were obtained from Sundaram Medical Foundation (SMF) hospital and Government General Hospital (GH), Chennai. Shiga-toxin producing diarrhogenic *Escherichia coli *O157 was obtained from Christian Medical College (CMC), Vellore. Reference strains of *Escherichia coli *and non-*Escherichia coli *organisms were obtained from Microbial Type Culture Collection (MTCC), Institute of Microbial Technology, Chandigarh. All the organisms were cultured in Luria-Bertoni (LB) broth at 37°C for 16-18 hours at 195 rpm. All clinical isolates were characterized biochemically followed by PCR using primer pairs targeting *fimH *gene of *Escherichia coli *[32] prior to validation by AuNP- oligo probe assay. The details of PCR cycling conditions were described in additional file 1. The colloidal gold nanoparticles of size 20 nm were purchased from Sigma and stored at 4°C until use. The silica spin column for DNA extraction was purchased from Qiagen. All other chemicals were of analytical grade and used as purchased without further modifications.

### Probe design

Probe sequence was designed based on the *malB *gene region that has the high homology among the *Escherichia coli *strains. A 20 base oligonucleotide was generated as probe sequence using the NCBI BLAST nucleotide search tool [33]. Care is taken in designing the sequence so that it does not share any sequence homology with non- *Escherichia coli *family members. Additionally, the melting temperature of the probes was ensured to be within a narrow range. Further the sequence is checked for potential self-complementarities and also formation of secondary structures was verified using mfold software [34] which may otherwise hinder the assay. The final 20 base probe sequence obtained was 5' TACAAAGGGAGAAGGGCATG 3'. It contains a thiol modifier at 5' end to enable conjugation with the colloidal gold nanoparticles. This 5' alkyl thiol modified oligonucleotide with HPLC purification was purchased from Sigma.

### Preparation of AuNP-oligo probe

The AuNP- oligo probe was synthesized using a previously described protocol [35]. Briefly, the 4 nmol thiol modified oligonucleotide was initially incubated with 1 ml of AuNPs overnight in an orbital shaker at room temperature wrapped in aluminium foil. At the end of reaction, phosphate buffer (100 mM, pH7) was added to obtain a final concentration of 9 mM. The surfactant solution containing sodium dodecyl sulphate was added resulting in a concentration of 0.1% and incubated in an orbital shaker for 30 minutes. The salting solution (2 M NaCl in 10 mM PBS pH7) was divided in six doses and added to the above solution over the course of the two days to reach a final concentration of 0.3 M NaCl. After the last salt addition the solution is allowed to equilibrate overnight at room temperature. Then centrifuged at 13000 xg for 20 minutes and the precipitate was washed with 500 μl of resuspension buffer containing 10 mM PBS (pH7.4), 150 mM NaCl, 0.1% SDS and resuspended in 50 μl of the same buffer. The fully functionalized AuNP- oligo probes retain the same color as the un-modified AuNPs with no visible aggregates, and stored in light-tight containers at room temperature until use.

### Genomic DNA isolation

The genomic DNA was isolated from the organisms using silica spin column as described elsewhere with slight modifications [36]. The organisms were inoculated in 10 ml of LB broth and grown overnight at 37°C in a shaker at 195 rpm. A two ml of culture was taken in a micro centrifuge tube and the pellet was collected after centrifugation at 6,000 xg for two minutes. The pellet was resuspended in 500 μl of lysis buffer (4.7 M guanidium thiocyanide, 46 mM Tris-HCl pH 6.4, 20 mM EDTA and 1.2 w/v% Triton X-100) and incubated at 37°C for 15 minutes. After incubation the cell lysate was transferred to column and centrifuged at 6,000 xg for three minutes. Collection tube was removed and the column washed with 650 μl of wash buffer (5.25 M guanidium thiocyanide and 50 mM Tris-HCl pH 6.4) at 6,000 xg for three minutes. Subsequently the column washed thrice with 70% ethanol followed by a dry spin at 6,000 xg for three minutes to remove any trace amount of ethanol in the column. Fifty microlitres of nuclease free water was added to the center of the column and incubated at room temperature for one minute and centrifuged at 10,000 xg for three minutes to elute the DNA. The concentration of genomic DNA was quantified at 260 nm using UV-VIS spectrophotometer (Cary 4000). The genomic DNA was stored at -20°C until use.

### AuNP- oligo probe hybridization assay

The complementary oligonucleotide (5'CATGCCCTTCTCCCTTTGTA3') specific to the probe sequence was obtained from Sigma. The complementary oligonucleotide from 100 μM stock was diluted (29.8 ng, 11.94 ng, 5.97 ng, 2.98 ng) in 10 mM PBS buffer (pH 5) to the final volume of ten microlitres. The DNA was heated to 95°C for five minutes and three microlitres of AuNP- oligo probe was added immediately followed by incubation at 65°C for ten minutes. Blank samples were prepared in exactly the same way using an equivalent volume of 10 mM PBS (pH5) instead of DNA. The negative control was prepared using non complementary DNA (5'GCCCTGACGAAGAAGGTGGC3') containing 61.9 ng of oligonucleotide in the final volume of ten microlitres. After hybridization the test sample was equally divided into two aliquots and subjected to gel electrophoresis and acid challenge simultaneously. The color of the solution was noted five minutes after the addition of 40 μl of 0.01 N HCl and photographed. The 1.5% agarose gel was used to demonstrate the hybridization of the probe with the complementary DNA. After electrophoresis the gel was stained for silver enhancement to visualize the migration of gold nanoparticles. In addition the oligonucleotide was chemically cleaved from the surface of the gold nanoparticles [35]. The retrieved oligonucleotide was hybridization with complementary DNA to conform the attachment of oligonucleotides to the surface of gold nanoparticles (See additional file 1).

### AuNP- oligo probe assay on genomic DNA

The hybridization protocol for complementary oligonucleotide was directly applied on *Escherichia coli *genomic DNA obtained from MTCC and found to be satisfactory for reliable detection. To determine the minimum quantity of DNA that can be detected using AuNP- oligo probe assay, ten microlitres of DNA in the concentration range of 215 ng, 108 ng, 54 ng and 27 ng was serial diluted from 430 ng of stock DNA in 10 mM PBS (pH5). Each dilution was tested in AuNP- oligo probe assay. Blank samples containing PBS instead of genomic DNA was used as control for visual comparison and characterized by UV-Vis spectrophotometer (Ocean Optics USB4000) to demonstrate the surface plasmon resonance shift in the samples. To determine the effect of pre-treatment of DNA on detection limit of the assay, genomic DNA was subjected to enzymatic digestion using EcoRV enzyme (New England Biolabs) as recommended by the supplier. Briefly, 57 ng of genomic DNA was mixed with 1 μl of 10× NEB buffer and 0.1 μl of 10 mg/ml BSA in the total volume of 10 μl. The above mixture was incubated at 37°C for 1 hour followed by 80°C for 20 minutes to stop the reaction. Also, same amount of genomic DNA was sonicated for five minutes in an ultrasonic bath (Branson 1510, 70 W, 42 KHz). In both the methods the DNA fragmentation were verified by agarose gel electrophoresis. The fragmented DNA samples were serially diluted (22.8 ng, 11.4 ng, 5.7 ng) and hybridized with AuNP- oligo probe. After hybridization, the reaction mixture was equally divided and subjected to acid challenge and gel electrophoresis.

### Evaluation on clinical isolates

We obtained 31 *Escherichia coli *clinical samples from patients with suspected enteric infections with known culture and biochemical based examination results, and we tested these isolates with AuNP-oligo probes based method. We analyzed 30 negative strains consisting of six samples in each of *Klebsiella pneumoniae, Pseudomonas aeruginosa, Proteus mirabilis, Salmonella Typhi *and *Salmonella ParatyphiA*. The source of organisms used for the assay was listed in Table 1. From each species, 100 ng genomic DNA is extracted through silica spin column method, denatured at 95°C for ten minutes and three microlitres of AuNP- oligo probes was added. To this sample, 0.01 N HCl added and results are recorded. To assess the repeatability of the method, testing with the proposed assay was repeated five times for each sample.

**Table 1:** Bacterial strains used for the evaluation of AuNP- oligo probe assay.

Organisms	Collection centre*	Number of isolates	PCR	Detection by AuNP-oligo probe assay
*Uropathogenic*	MTCC, IMTECH	1	+	+
*Escherichia coli*	SMF Hospital	20	+	+
(UPEC)	GH Hospital	10	+	+

*Escherichia coli O157*	CMC, Vellore	1	+	+

*Klebsiella pneumonia*	MTCC, IMTECH	1	_	_
	SMF Hospital	5	_	_

*Pseudomonas aeruginosa *	MTCC, IMTECH	1	_	_
	SMF Hospital	5	_	_

*Proteus mirabilis*	MTCC, IMTECH	1	_	_
	SMF Hospital	5	_	_

*Salmonella Typhi*	MTCC, IMTECH	1	_	_
	SMF Hospital	5	_	_

*Salmonella Paratyphi A*	MTCC, IMTECH	1	_	_
	SMF Hospital	5	_	_

*SMF- Sundaram Medical Foundation Hospital, Chennai. GH- Government General Hospital, Chennai. CMC- Christian Medical College, Vellore. MTCC- Microbial Type Culture Collection. IMTECH- Institute of Microbial Technology, Chandigarh.

### Preparation of spiked urine samples

The urine from normal person was spiked with uropathogenic *Escherichia coli *isolated from urinary tract infected patient. To prepare urine spiked clinical samples, 10^6 ^- 10^5 ^cfu/ml *Escherichia coli *were added to one ml of urine and centrifuged at 14000 xg for 25 minutes. The supernatant was discarded and the pellet was used for genomic DNA extraction as described above. The genomic DNA was quantified and the average yield was determined to be 50-60 ng. About 30 different clinical (spiked) samples were independently assayed using AuNP- oligo probe assay and by PCR. The details of the PCR conditions were described in the additional file 1. Subsequently to demonstrate the specificity of the assay, the other major uropathogens namely, *Klebsiella pneumonia, Pseudomonas aeruginosa *and *Proteus mirabilis *were also spiked in urine as described above and the extracted genomic DNA was used for the AuNP- oligo probe assay.

### Imaging by Atomic Force Microscopy

AFM imaging was performed to examine the nature of positive and negative samples under test condition. Sample slides for AFM were made using freshly cleaved mica sheets soaked in poly-l-lysine and incubated at room temperature for 20 minutes. The excess poly-l-lysine was washed with distilled water. Slides are baked at 55°C for one hour. The sample for AFM imaging was prepared on these slides as described previously with slight modifications [37]. Briefly, the test samples after completion of assay were centrifuged at 13000 xg for 30 minutes and the pellet was washed twice in distilled water to remove salt and unhybridized DNA. The pellet was resuspended in 50 μl of distilled water and spread over the poly-l-lysine coated mica sheets and incubated at room temperature for 20 minutes. The excess samples were removed and mica sheets were washed twice with distilled water and allowed to air dry at room temperature. The images were obtained using Scanning probe microscope (NanoscopeIII, Digital Instruments, Santa Barbara, CA, USA) in contact mode on the air using silicon nitrite tips.

## Results and Discussion

### Characterization of AuNP- oligo probe assay

**Figure 2:**
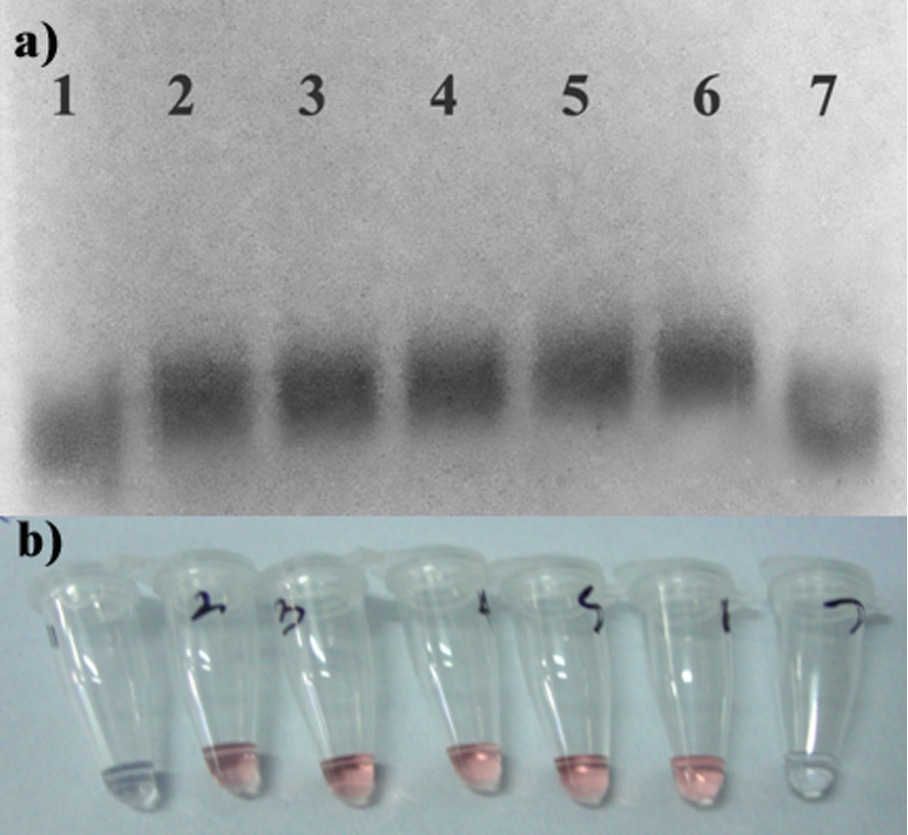
**a) Silver enhanced agarose gel showing AuNP- oligo probe hybridization**. Lane 1: AuNP- oligo probe alone. Samples with increasing concentration of complementary DNA (Lane 2: 2.8 ng, Lane 3: 5.6 ng, Lane 4: 11 ng, Lane 5: 28 ng, Lane 6: 59 ng). Lane 7: 61.9 ng of non complementary DNA. b) Corresponding results recorded visually after acid challenge.

Initially, the AuNP- oligo probe assay was optimized for visual detection using 20 base complementary sequences. It is noted that annealing at 65°C was determined to be the optimum temperature for hybridization of AuNP- oligo probes and the target DNA. The complementary DNA after hybridization with the AuNP- oligo probe was separated by gel electrophoresis and subjected to silver enhancement (Figure 2a). The gradual decrease in migration of the gold nanoparticles was observed with increase in concentration of the complementary DNA in the sample (Lane 2-6) when compared to the migration of AuNP- oligo probe (Lane 1) and the negative control (Lane 7). Thus hybridization was revealed by shift in gold nanoparticle band towards lower electrophoretic mobility. This in fact acts as an evidence for conjugation of the oligonucleotide to the surface of the gold nanoparticles. In turn the hybridization was evident from the mobility-shift proportionate to the concentration of the complementary DNA. In Figure 2b no visual discrimination of color change from red to purple was observed up to 2.8 ng of complementary DNA. Whereas the unhybridized AuNP-oligo probe loses its stability and tends to aggregate upon acid addition. Expectedly, non complementary DNA exhibited aggregation upon acid addition with visual change in color of the solution from red to purple. This initial validation in fact confirmed the AuNP- oligo probe hybridization with the target DNA present in test sample.

### AuNP- oligo probe assay for *Escherichia coli*

**Figure 3:**
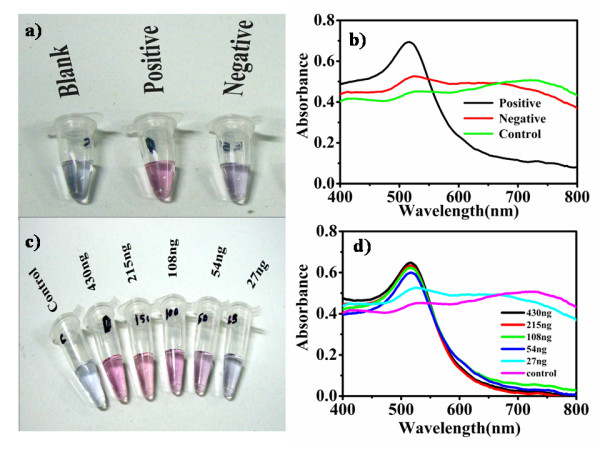
**a) Representative results recorded visually after acid addition**. Blank, positive, and negative denotes sample tubes containing PBS, genomic DNA of *Escherichia coli *and *Pseudomonas aeruginosa *respectively. Red color shows the presence of target and purple indicates the absence. b) UV-Vis spectra of the respective samples. c) Visual observation of minimum detection limit of AuNP- oligo probes hybridized with serial dilutions of *Escherichia coli *genomic DNA in 50 μl volume. d) UV-Vis spectra of the respective samples showing characteristic absorption at 520 nm upto 54 ng DNA and broad spectral shift for samples containing less than 54 ng DNA and control sample.

The AuNP- oligo probe assay is applied to the genomic DNA isolated from *Escherichia coli*. Figure 3a shows results of visual discrimination of positive and negative samples along with control sample having PBS instead of DNA. It is observed that the solution containing *Escherichia coli *genomic DNA remains red, whereas the solution containing

*Pseudomonas aeruginosa *genomic DNA and the control turned purple after addition of 0.01 N HCl (Figure 3a).

As outlined in Figure 1, hybridization of AuNP- oligo probe with the target DNA formed double stranded DNA helix that stabilizes the gold nanoparticles under acidic environment and hence remains red. However, in the absence of complementary DNA the AuNP- oligo probes aggregate under acidic environment and the solution turns purple. UV-Vis spectroscopic data for these samples given in Figure 3b support the hypothesis of aggregation induced visual discrimination of the sample. It is seen that sample containing *Escherichia coli *genomic DNA showed characteristic absorbance peak of AuNP at 520 nm (black line) due to collective excitation of the free conduction band electrons of the dispersed particles known as the surface plasmon resonance. Whereas in the negative (red line) and control (green line) samples exhibiting wide absorbance spectrum is indicative of peak shift towards longer wavelength (≥600 nm) due to the coupling in the surface plasmons of the particles in the aggregates. This behaviour is in conformity to the theoretical descriptions of Mie scattering from similar small aggregate clusters where the plasmon resonance absorption of the aggregates give rise to additional long wavelength component in the optical absorption spectrum relative to the absorption spectra of dispersed nanoparticles in the solutions [38]. The observed pale blue to purple color solution is also in agreement with this understanding.

### Limit of detection

Various dilutions of *Escherichia coli *genomic DNA was used to determine the limit of detection of the AuNP- oligo probe assay. Figure 3c summarizes the results of detection sensitivity obtained visually after acid addition. It was observed that no visible change in color of the solution occurred up to sample containing ~54 ng of *Escherichia coli *genomic DNA below which the solution turned purple indicating the aggregation of the AuNP- oligo probes. All the samples were subsequently measured for their absorbance to evaluate the surface plasmon resonance peak shift respective to the presence of different concentration of target DNA in the test sample. Figure 3d shows the dependence of the absorption spectra of the AuNP- oligo probe on the target DNA concentration ranging from 430 to 27 ng of genomic DNA compared with the control sample. All the positive samples were found to have the characteristic absorbance peak at 520 nm with gradual decrease in absorbance relative to the concentration of genomic DNA present in the test sample. Whereas in the negative and control samples exhibited the red shift in their spectra with the absorbance peak shifted to longer wavelength (≥600 nm) whereas there was considerable peak decrease in 520 nm. Thus AuNP- oligo probe was found to detect the presence of ~ 54 ng of genomic DNA of target pathogen after acid induced aggregation defining the minimum detection limit of the assay.

**Figure 4:**
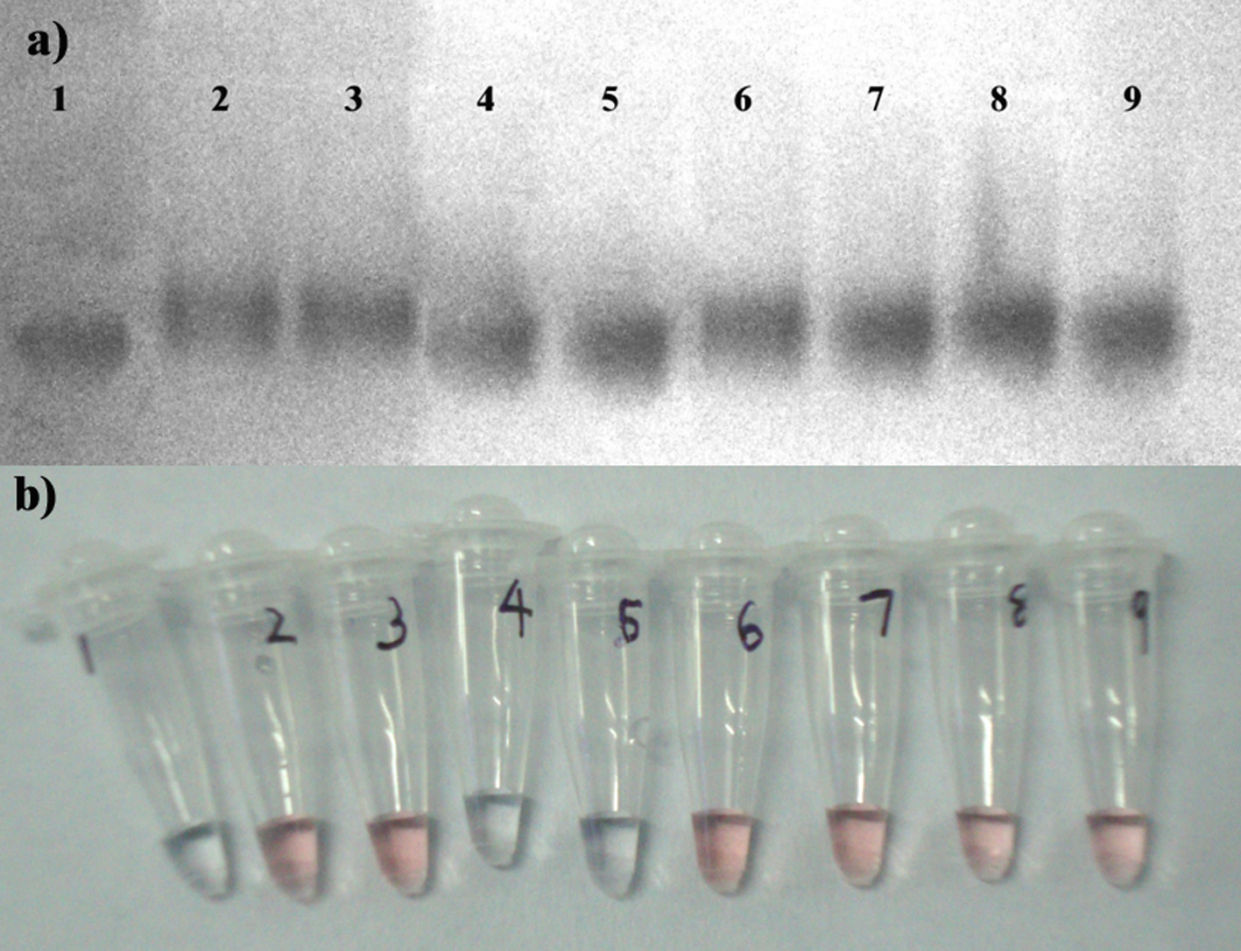
**a) Hybridization of AuNP- oligo probe with genomic DNA**. Lane 1: AuNP- oligo probe. Lane 2-3: Hybridization with 22.8 ng and 11.4 ng of restriction digested DNA respectively. Lane 4-5: Hybridization with 22.8 ng and 11.4 ng of sonicated DNA respectively. Lane 6-9: Hybridization with 430 ng, 215 ng, 108 ng, 54 ng of untreated genomic DNA respectively. b) Corresponding results recorded visually after acid challenge.

To further increase the detection limit of the assay, the genomic DNA was pre-treated prior to use in the assay and compared with that of untreated genomic DNA. Figure 4 summarizes the results of detection limit by gel electrophoresis and visual detection.

As observed in Figure 4a, the unhybridized AuNP- oligo probe moved a longer distance (Lane1) compared to the probe hybridized with the restriction digested DNA (Lane 2-3) and sonicated DNA (Lane 4-5). To note, the progressive retardation in the shift of the gold nanoparticle band was observed with increasing concentration of genomic DNA in the test sample (Lane 6-9). This result is in agreement with the result of concentration dependent hybridization of AuNP- oligo probe with the complementary DNA (Figure 2). In this way, we believe that gel electrophoresis is a very powerful method to investigate the hybridization of target sequence to the AuNP- oligo probe. Thus specific and non specific attachment of DNA can be detected with high sensitivity. The representative results observed visually after acid addition was summarized in Figure 4b. However the AuNP- oligo probe failed to show increase in the detection sensitivity of the assay on sonicated genomic DNA, attributed to the fact that sonication may lead to random shearing of DNA with many single stranded nicks. These data in total suggest that restriction digestion of genomic DNA can efficiently improve the detection limit of the assay from ~54 ng to 11.4 ng.

### Evaluation of specificity on clinical isolates

**Figure 5:**
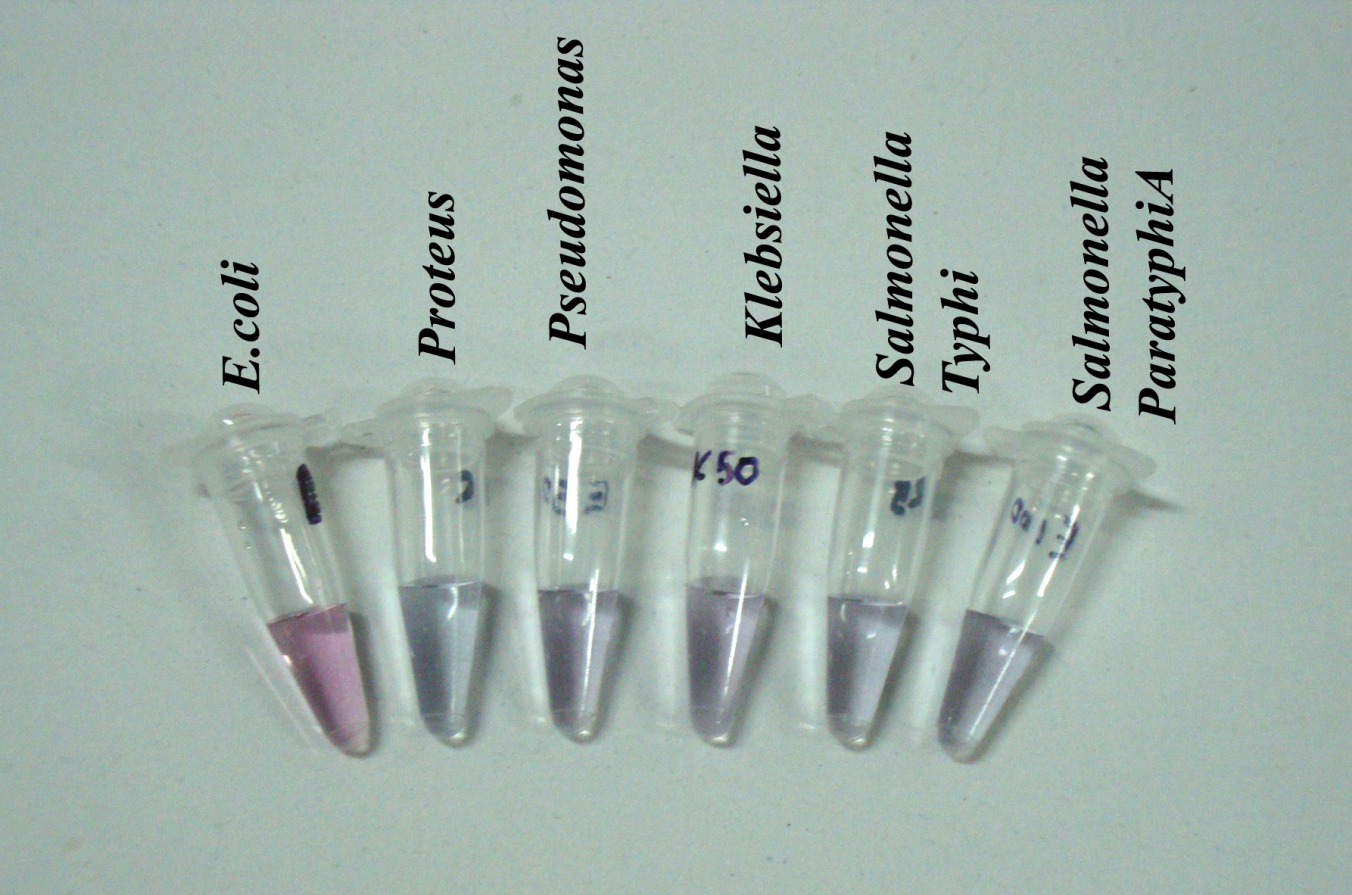
**Representative snap shot of AuNP- oligo probe assay on DNA samples of indicated bacterial species**. *Escherichia coli *sample exhibit red color confirming the presence of target DNA. All other samples exhibit purple color suggesting the absence target DNA.

In order to obtain an indication of the methods performance on clinical samples, we applied the optimized assay on DNA isolated from the bacterial species listed in Table 1. In total 31 *Escherichia coli *clinical samples were tested in the assay and were found to be true positives. During the evaluation we found that all true negatives in the AuNP- oligo probe assay was negative controls confirmed in our laboratory by PCR. Figure 5 gives representative snap-shot of visual observations of various negative controls compared with *Escherichia coli *strain exhibiting visible color change of the solution from red to purple after acid addition. Thus the discrimination of *Escherichia coli *and non- *Escherichia coli *strains were accomplished visually without the need of any sophisticated instrument. The application of the proposed method on the DNA isolated from the clinical isolates produced exactly the same results every time (n = 5) when the assay was repeated. Hence the assay defines to have 100% specificity and repeatability thus enabling reliable and highly specific detection without any cross reaction with related bacteria. An interesting feature of this assay is that the positive samples when preserved overnight at room temperature continues to retain its color suggesting the long-time stability of AuNP- oligo probe hybridization with the target sequence. This feature is particularly useful for prolonged read-out capability required for high-throughput applications.

### Validation on clinical samples

The genomic DNA extracted from spiked urine samples were used for AuNP- oligo probe assay. The assay detected all 30 urine spiked uropathogenic *Escherichia coli *strains with zero cross reactivity against closely related uropathogens namely, *Pseudomonas aeruginosa, Klebsiella pneumoniae *and *Proteus mirabilis*. Independently, PCR was performed on these entire samples with same quantity of genomic DNA used for AuNP- oligo probe assay (See additional file 1). The primer pair specific for detection of uropathogenic *Escherichia coli *were used as described elsewhere [32]. All samples gave specific amplicons without any discrepancy with the results of AuNP- oligo probe assay. Usually the diagnosis of urinary tract infection was based on a quantitative urine culture yielding greater than 10^5 ^cfu/ml of urine, which was termed significant bacteriuria. This value was arrived because of its high specificity to the diagnosis of true infection, even in asymptomatic persons [39]. The range of detection of our AuNP- oligo probe assay is well within this limit. We further show that pre-treatment of genomic DNA with restriction digestion can fivefold increase the detection limit on the real clinical samples. Therefore this assay proves to be an efficient alternative to conventional culture based detection. Thus a rapid method for identifying uropathogenic *Escherichia coli *directly from clinical sample has been developed. We note that 100% positive results were obtained every time (n = 3) when using AuNP- oligo probes prepared in three batches spread over a period of six months. This proves the repeatability of the assay and the robustness of AuNP- oligo probe.

### AFM Characterization

**Figure 6:**
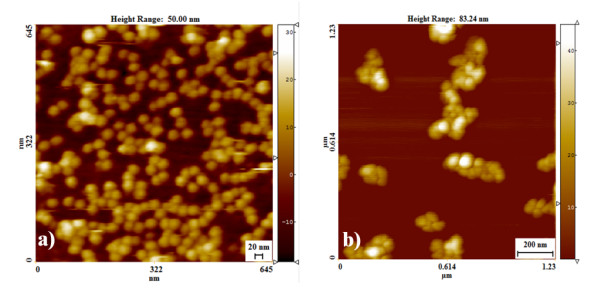
**AFM image of AuNP- oligo probe assay**. a) Monodispersed gold nanoparticles in the presence of *Escherichia coli *genomic DNA in the test sample. b) Three dimensional aggregates of gold nanoparticles in the presence of *Pseudomonas aeruginosa *genomic DNA in the test sample.

High resolution images of nanoparticles by AFM have been successfully used in the past to study of DNA- nanoparticle interactions [23,37,38,41]. To elucidate the nature of AuNP- oligo probe and target DNA interaction in our test samples, AFM images of these complexes are taken. Figure 6a shows the representative image of AuNP- oligo probe hybridized with *Escherichia coli *genomic DNA (positive samples). Interestingly, there is no evidence of gold nanoparticles growth as the hybridized probes seem to be remarkably regular in size with an average diameter of 20 nm. Note that fine monodispersed gold nanoparticles were observed despite large concentration of sample immobilized on the substrate surface. The results suggest that the nanoparticles remain separated from one another without aggregation. In sharp contrast, large scale aggregation of gold nanoparticles is observed in the presence of *Pseudomonas *genomic DNA, a non-complementary target, in the test sample (Figure 6b). We imply that single stranded probe oligonucleotide on the surface of gold nanoparticles could not prevent them from aggregation under acidic condition. Therefore, it can be concluded that the formation of nanoparticle aggregates is indeed due to the absence of complementary DNA in the solution. This result further explains data on characteristic absorption spectra of positive and negative samples shown in Figure 3b. The optical property of gold nanoparticle studied by UV-Vis spectroscopy gave quantitative information about the degree of aggregation, indicating whether the particles are well separated from one another or whether they predominantly form aggregates. This optical property qualitatively correlates with the structural features studied by AFM.

In summary, we have elucidated the utility of AuNP- oligo probe as a signalling probe for rapid detection of *Escherichia coli*. Though there exist many methods using AuNPs for detection such as scanometric [42], surface enhanced Raman scattering [43] and electrochemical techniques [44], AuNPs based colorimetric detection continue to hold great potential in point of care based applications. Most useful attributes of this system is the fact that AuNP- oligo probes can maintain good dispersion in solution and posses shelf-life more than six months at room temperature. Using UV-Vis spectroscopy and AFM imaging, we have quantitatively demonstrated accuracy and reliability of visual detection method. Striking feature of this protocol is the requirement of very low concentration of genomic DNA for detection. We emphasize that even though molecular detection methods like PCR and a more recent technology of real-time PCR constitute robust and sensitive technique, it requires sensitive equipment and trained personnel. The salient feature of the methodology developed here combines the gold standard of DNA based detection with a simplified and user-friendly protocol for point-of-care application. An important goal for improved diagnosis is the development of rapid and accurate method for detection of bacterial pathogens. Most current clinical methods that identify bacterial strains require time-consuming culture of the sample or procedures involving polymerase chain reaction. Neither of these approaches has enabled testing at the point -of-need because of requirement of skilled technicians and laboratory facilities. We believe this approach could hold potential as next generation diagnostic tools. This is a significant advance in DNA based diagnostics.

## Conclusion

In this paper, an ultrasensitive and rapid DNA detection technique based on AuNPs functionalized with single stranded oligonucleotide probe was demonstrated. This AuNP- oligo probe strategy allowed for visual detection of *Escherichia coli *from its genomic DNA of the order of 11.4 ng when the genomic DNA was pre-treated by restriction digestion. AFM and UV-Vis spectroscopic investigations give evidence to the formation of gold nanoparticle aggregates in the solution. To demonstrate the relevance of the methodology for clinical diagnostics, extensive validation was performed on clinical (spiked) samples. The results demonstrate the assay to be highly sensitive and reliable screening tool for rapid detection of *Escherichia coli *causing urinary tract infection. The detection of presence of bacteria could be performed with high sensitivity and specificity within the turnaround time of 30 minutes. We emphasize that the clinically relevant detection limit of ≥10^5 ^cfu/ml for having high specificity on asymptomatic persons is easily met in our assay. We believe that use of nanoparticles based detection has pushed the measurement sensitivity level comparable to those demonstrated by traditional molecular biology methodologies. Finally, the proposed method represents a generic gold nanoparticle based platform technology that has the potential point of care application for clinical detection of bacterial pathogens. They open a further possibility of designing new devices with their unique local optical properties.

## List of Abbreviations

(AuNPs): gold nanoparticles; (AuNP- oligo probe): AuNP conjugated oligonucleotide probe.

## Competing interests

The authors declare that they have no competing interests.

## Authors' contributions

BP carried out the conjugation of the nucleotide to the gold nanoparticles, probe sequence designing, participated in the design of the study, and performed the detection assay and participated in the drafting of manuscript. RVK carried out the UV-Vis spectroscopic measurements, preparation of samples for AFM imaging and participated in the AFM imaging. BMJA conceived the experiment, participated in design and coordination and drafted the manuscript. All authors read and approved the final manuscript.

## Authors' information

BP is a Ph.D. scholar working on development of rapid detection protocols for bacterial pathogens as her main topic of research thesis. RVK is a research associate in the laboratory of BMJA in a sponsored research project. RVK has considerable research experience in device development. BMJA is a thesis supervisor for BP. BMJA has wide experience in the development of biosensors and optical manipulation of biomolecules for biophysical investigations. BMJA after serving as Faculty Scientist at AU-KBC Research Centre for eight years has moved to Pondicherry University as a Reader at Centre for Green Energy Technology. His current research includes microbial source of hydrogen energy production, biosolar cells device development.

## Supplementary Material

Additional file 1 **Characterization and validation of AuNP- oligo probe assay**. Hybridization of complementary DNA with chemically cleaved oligonucleotide retrieved from AuNP- oligo probe. Detection of urine spiked clinical samples by PCR. Click here for file
