# Supplementary material for: A direct detection of Escherichia coli genomic DNA using gold nanoprobes
Source: J Nanobiotechnology. 2012 Feb 6;10:8. doi: 10.1186/1477-3155-10-8 (PMC3306828; doi:10.1186/1477-3155-10-8)
Supplement: Additional file 1 — Characterization and validation of AuNP- oligo probe assay. Hybridization of complementary DNA with chemically cleaved oligonucleotide retrieved from AuNP- oligo probe. Detection of urine spiked clinical samples by PCR. [file 1477-3155-10-8-S1.PDF]

Additional file 1

A direct detection of *Escherichia coli* genomic DNA using gold nanoprobes

Bakthavathsalam Padmavathy [1], Rajendran Vinoth Kumar [1], Baquir Mohammed Jaffar Ali.[2,*]

Materials and methods

Characterization of AuNP- oligo probe

In order to prove the conjugation of oligonucleotides to the surface of the gold nanoparticles, the DNA was chemically displaced from the nanoparticle surface using DTT [35]. The equal volumes of AuNP- oligo probe and 1 M DTT in 10 mM Phosphate buffer pH 7.4 was incubated at 50°C for 15 minutes and then 45 minutes at 25°C. The oligonucleotides were released into solution during incubation and the gold nanoparticles precipitate with change in color of the solution from red to purple. The supernatant containing the released DNA was removed after centrifugation leaving the gold nanoparticle. The supernatant was incubated with the complementary DNA at 65°C for ten minutes and the mixture was subjected to gel electrophoresis to demonstrate the hybridization.

PCR on clinical samples

The genomic DNA from clinical isolates and clinical samples was isolated by silica spin column method as described above. The PCR master mix and nuclease free water was purchased from Fermantas Life sciences. The primer pair targeting the Type I fimbriae

gene (*fimH*) of *Escherichia coli* was adopted from previous publication as described elsewhere [32]. The forward primer 5' TCGAGAACGGATAAGCCGTGG3' and reverse primer 5'GCAGTCACCTGCCCTCCGGTA3' resulting in the amplicons size of 508bp was used for the reaction. The PCR amplification was carried out in total reaction volume of 25µl containing 12.5 µl of 2X PCR master mix (10 mM Tris-HCl, 50 mM KCl, 1.5 mM $MgCl_2$, 0.2 mM dNTPs, 5 % Glycerol, 0.08 % NP-40, 0.05 % Tween-20, 25 units/ml *Taq* DNA Polymerase pH 8.6), 1µM final volume of forward and reverse primer, 2µl of genomic DNA and desired amount of nuclease free water to make up to the final volume. The amplification was carried out at 95°C for five minutes followed by 35 cycles at 95°C for 50 seconds, 54°C for 55 seconds, 72°C for 50 seconds and final extension at 72°C for ten minutes to complete the synthesis of all strands. The PCR was carried out in thermal cycler (Minicycler™ MJ Research). Following the amplification, 6 µl of amplicon were separated on 2.5% horizontal agarose gel for one hour at 100V. The amplicons were visualized with a UV transilluminator and photographed. The size of the amplicons was determined using 50bp molecular size marker.

Results

AuNP- oligo probe hybridization

The chemically retrieved oligonucleotide from AuNP- oligo probe prepared on three different occasion spread over a period of six months were incubated with complementary DNA and separated by agarose gel electrophoresis. As observed in Figure 1, the hybridization with the complementary DNA was confirmed (Lane1-3)

against the control sample containing probe sequence and complementary DNA (Lane 4).

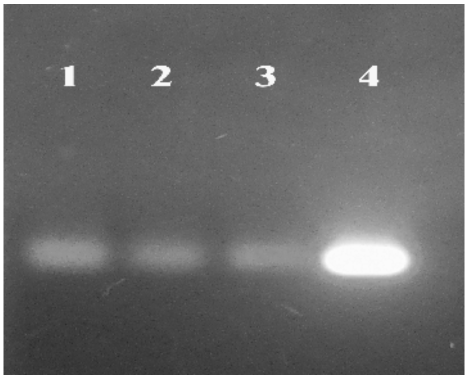

Figure1. Hybridization of complementary DNA with chemically retrieved oligonucleotide from three independent AuNP- oligo probe (Lane 1-3). Lane 4: Control reaction showing hybridization of probe sequence with the complementary DNA.

PCR on clinical samples

About 30 clinical isolates and clinical (spiked) samples were subjected to PCR. The representative results of PCR was summarized in Figure 2 showing the specific 508 bp amplicons on the gel stained with ethidium bromide. The size of the amplicons was determined using 50bp molecular size marker.

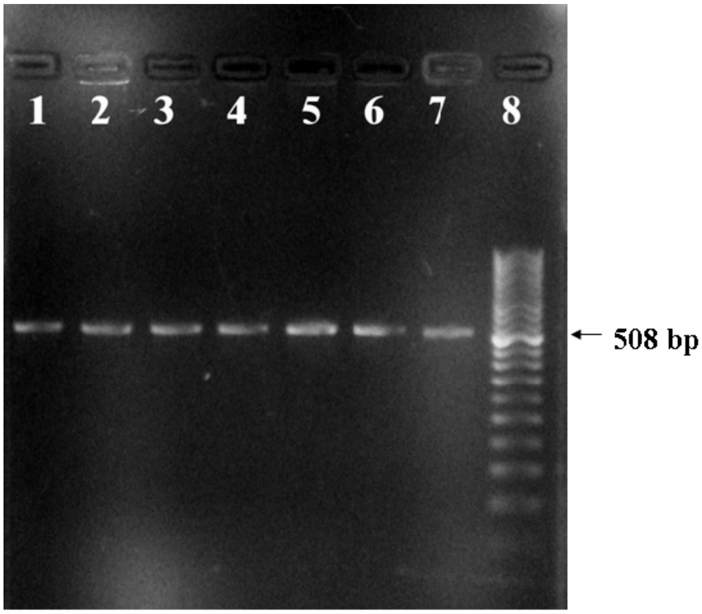

Figure2. Detection of urine spiked clinical samples by PCR. Lane 1-7: Visualization of 508 bp amplicons specific to uropathogenic *Escherichia coli.*
